# Cats just want to have fun: Associations between play and welfare in domestic cats

**DOI:** 10.1017/awf.2023.2

**Published:** 2023-01-27

**Authors:** Julia Henning, Torben Nielsen, Eduardo Fernandez, Susan Hazel

**Affiliations:** School of Animal and Veterinary Sciences, The University of Adelaide, Mudla Wirra Rd, Roseworthy SA 5371, Australia

**Keywords:** animal welfare, behaviour, cat, human-animal interaction, play, quality of life

## Abstract

Play is often considered an indicator and promotor of animal welfare and may facilitate closer cat-human relationships. However, few studies have empirically investigated these associations. The current study aimed to investigate play-related factors associated with four welfare outcome measures in cats (*Felis catus*) including: cat quality of life; cat-guardian relationship quality; problem behaviour prevalence; and behavioural changes. An online survey was developed using demographic information, questions related to play and resources, free text sections and the following validated measures: cat quality of life (QOL), the cat owner relationship scale, and the adult playfulness trait scale. Responses were completed by 1,591 cat guardians from 55 countries. Higher cat playfulness scores and a greater number of games played were significantly associated with higher cat QOL scores while longer amounts of daily play, greater number of games, both cat and guardian initiating play and higher guardian playfulness scores were all significantly associated with higher cat-guardian relationship scores. Exclusively indoor housing was significantly associated with both higher cat QOL and higher cat-guardian relationships scores compared to cats with outdoor access. Behavioural changes associated with distress in cats were reported when play was absent. Play may be an important factor in assessing and maintaining cat welfare. Further research into the mechanisms of how play impacts welfare and cat-guardian relationships is needed.

## Introduction

The potential of play as a behavioural tool to both identify and increase animal welfare has been a recent topic of interest for applied animal behaviour researchers (Held & Špinka 2011; Ahloy-Dallaire *et al*. 2018). This link between play and welfare is most often observed when animals are experiencing good health, have adequate resources and are free from fitness threats such as predation (Fagen 1978; Oliveira *et al*. 2010). In addition, research links play to juvenile development and long-term maintenance of neurological/physiological, cognitive-behavioural, and emotional skills (Burghardt 2005; Graham & Burghardt 2010; Vanderschuren & Trezza 2013; Pellis *et al*. 2015).

Play may be especially useful in managing the welfare of animals in human care such as cats (*Felis catus*), an increasingly popular pet choice within human homes (PDSA Animal Wellbeing Report 2022). Previous studies into play in cats have found associations between welfare issues such as social isolation, inconsistent husbandry, space availability, problem behaviours and changes in play behaviour (Seitz 1959; West 1974; Guyot *et al*. 1980; Carlstead *et al*. 1993; Strickler & Shull 2014; Loberg & Lundmark 2016). Of the previous studies that assessed play and welfare associations in cats, only a few used a specific welfare metric within their study. For instance, measures of cat physical condition (Arhant *et al*. 2015), occurrence of abnormal, repetitive behaviours (Kogan & Grigg 2021) or physiological and behavioural signs of stress (Carlstead *et al*. 1993). In a recent review of the current literature, we highlighted the lack of specific welfare measures used in studies of play in cats (Henning *et al*. 2022a).

Cat welfare encompasses many elements, from the cats’ physical state and the quality of their environment as well as the resources available to them, to their mental state and social relationships (Ellis *et al*. 2013; Foreman-Worsley & Farnworth 2019; Henning *et al*. 2022a). For cats in human homes, all these elements rely heavily on their guardian and their relationship with that guardian. It is therefore important to consider guardian perceptions about their cat, their cat’s behaviour and their relationship with their cat when assessing cat welfare within a human home. Human-animal interactions, such as play, are likely to impact the dynamic and quality of relationships between cat and guardian. Considering play may be integral to forming and maintaining social skills and communication intra-specifically in animals (Guyot *et al*. 1980, Bekoff & Allen 2011; Vanderschuren & Trezza 2013; Palagi *et al*. 2016), play may also be capable of assisting in establishing and maintaining healthy cat-guardian relationships (Henning *et al*. 2022b).

Guardians are responsible for making homing and medical choices for their cats which may have serious welfare outcomes, such as surrender or euthanasia. How guardians perceive their cats may impact how they treat them and even what decisions they make concerning their lives. What guardians perceive as ‘problem behaviours’, such as scratching furniture or inappropriate urination are often the result of species-typical behaviours that are natural and functional within their usual environment but become problematic when viewed through the lens of a guardian who wishes to have a neat and undamaged home. Regardless of whether the behaviours are problematic for the cat or not, problem behaviours are the leading cat-related factor for surrender to shelters (Patronek *et al*. 1996; Jensen *et al*. 2020) and the foremost cause of euthanasia in otherwise healthy pet cats (Carney *et al*. 2014). Studies suggest that play may have a role in mitigating the occurrence of problem behaviours in cats, with a lack of play associated with greater occurrence of problem behaviours (Strickler & Shull 2014; Foreman-Worsley & Farnworth 2019).

While previous studies show promise for some association between play and welfare in cats, there is still much to be understood about whether play is an adequate indicator of welfare, whether play can be used to promote welfare and how much or what kind of play is best suited to achieving these aims. A previous study by Henning *et al*. (2022b) investigated the factors associated with how much play occurs within human-cat dyads. Here, the present study follows on from this paper and aims to identify and assess whether play itself, its quantity or quality or its role in cat-guardian relationships, is associated with welfare outcomes in cats. To achieve this, four different potential welfare measures were used within a global online survey. These were: cat quality of life (QOL) scores; quality of the cat-guardian relationship scores; reported behavioural problems; and guardian observations of behavioural changes in times when play was absent. It was expected that higher play times would be positively associated with welfare measures.

## Materials and methods

The survey development, validated measures, data management and study population have been described in more detail in Henning *et al*. (2022b). A short summary is included here as well as details relevant to the areas of the questionnaire analysed within this paper. The protocol for this study was conducted with approval from the Human Research Ethics Committee at the University of Adelaide, approval code: H-2021-091.

### Survey

The survey was developed in consultation with veterinarians, animal behaviourists, and cat guardians. Participants were required to be over 18 years of age and the primary caregiver of a cat over one year of age. If the participant had multiple cats, they were asked to think of the cat they spent the most time with and answer questions as if they had been asked about that cat specifically, so that the survey was only answered for one cat per respondent. The resulting open survey comprised 105 questions and was hosted on Qualtrics XM®. The survey included demographic information, questions regarding duration and time of play, play type, guardian experiences of play, cat medical history, guardian perceptions of changes in behaviour, and three validated measures which included: the Feline Quality of Life Measure (QOL) (Tatlock *et al*. 2017); the Cat Owner Relationship Scale (CORS) (Howell *et al*. 2017); and the Adult Playfulness Trait Scale (APTS) (Shen *et al*. 2014). All included measures had previously been validated (Shen *et al*. 2014; Howell *et al*. 2017; Tatlock *et al*. 2017). Participants were asked to rate whether their cat exhibited certain problem behaviours on a 5-point Likert scale from ‘never’ to ‘most of the time.’ Only participants who completed every question of the cat QOL and CORS validated measures, and completed at least 98% of the survey overall, were included in final analysis (Henning *et al*. 2022b). The survey was open for participation between the 22^nd^ of June and the 17^th^ of July 2021.

### Analysis

A Kolmogorov-Smirnov test was undertaken to assess normality. The data set were found not to be normally distributed. However, as the data set were large, and thus robust enough, to be tested using standard parametric inferential statistics, these were used (Ghasemi & Zahediasl 2012; Hector 2021). Eighteen reported problem behaviours were reduced to seven components using a Principal Component Analysis (PCA). After viewing the matrix, one component and one item were removed due to low loading, leaving six components and seventeen items. Components were then included in analyses, including ANOVAs, regression, and general linear models. Two general linear models were created with cat QOL and CORS scores as dependent variables. All variables were tested through two-way ANOVA against QOL and CORS scores, respectively, and all variables from regressions or ANOVAs with a *P* < 0.2 were included in subsequent modelling. Non-significant factors were removed using backwards elimination. Interactions that were relevant were checked for significance and included in the model where found. Statistical analyses were completed using SPSS® Statistics 27. A *P*-value of < 0.05 was considered significant. Descriptive and qualitative analyses of behaviour changes during times play was withheld were undertaken. Free text responses were collected and grouped into types of behaviour. Where quotes have been shortened a ‘[…]’ is used. Quotes were only shortened where necessary while maintaining and respecting the original meaning.

## Results

A total of 1,591 completed responses were recorded from 55 different countries. Most participants were currently living in Australia (49.3%), identified as female (90.3%) and reported living in a two-person household at the time of the survey (47.8%). Most cats in the study were of mixed breed (76%) and almost equally reported to be male or female. Most cats were reported to be housed exclusively indoors (67%), between 3–5 years of age (28.8%), within a single cat home (38.9%), and had been living with their current guardian for 2–5 years at the time of the survey (42%). For further information on demographics of this study see Henning *et al*. (2022b).

### Factors associated with problem behaviours

A list of problem behaviours and a free text option were presented to respondents along with a five-point Likert scale with options from ‘never’ to ‘most of the time.’ Answers above a three on the Likert scale were included in the following tallies and percentages. The most reported problem behaviour by participants was scratching furniture (47.5%), followed by aggression during play (40.2%), excessive vocalisation (37.6%), aggression towards unfamiliar cats (36.6%) and being overly active at night (35.8%) (Table 1).

**Table 1.** Problem behaviours reported by cat guardians (n = 1,591). Guardians were able to report multiple behaviours. Based on online survey responses of guardians between June 22^nd^ and July 17^th^, 2021

Problem behaviours reported	*n*	%
Scratching furniture	755	47.5%
Aggression during play (play aggression)	639	40.2%
Excessive vocalisation	598	37.6%
Aggression towards unknown cats	583	36.6%
Overly active at night	570	35.8%
Anxiety	424	26.6%
Aggression towards familiar household cats	424	26.6%
Aggression towards unknown animals	358	22.5%
Urinating outside of the litter-box	311	19.5%
Sucking on material	244	15.4%
Aggression towards other household animals	217	13.6%
Pica	206	12.9%
Overgrooming	186	11.7%
Aggression towards human household members	171	10.7%
Defaecating outside the litter-box	153	9.6%
Aggression towards unknown people	129	8.1%
Spraying	67	4.2%
Urinating and/or defaecating on furniture	59	3.7%

Eighteen items of reported problem behaviours were reduced using a PCA. Six components presented with an eigenvalue exceeding 1, explaining 51.6% of variance. Sucking on material did not present with a high enough value in any component and was removed from the analysis, leaving seventeen items. These summary indices included the following components: inappropriate excretion subset, aggression towards unknown animals’ subset, annoyance behaviour subset, aggression towards people subset, stress behaviour subset, and aggression towards known animals’ subset (Table 2).

**Table 2.** Loading for 18 problem behaviours reported by cat guardians, generated by means of a Principal Component Analysis

	Summary indices					
Problem behaviour	Inappropriate excretion	Aggression towards unknown animals’	Annoyance behaviour	Aggression towards people	Stress behaviour	Aggression towards known animals
Urinating outside of the litter-box	0.795					
Defaecating outside the litter-box	0.480					
Spraying	0.663					
Urinating and/or defaecating on furniture	0.788					
Aggression towards unknown cats		0.865				
Aggression towards unknown animals		0.865				
Scratching furniture			0.633			
Overly active at night			0.761			
Excessive vocalisation			0.586			
Aggression to household members				0.838		
Aggression to unknown people				0.744		
Aggression during play (play aggression)				0.349		
Overgrooming					0.606	
Pica					0.374	
Anxiety					0.672	
Sucking*					0.052	
Aggression towards familiar household cats						0.709
Aggression towards other household animals						0.697

* Not included due to low loading

Analysis of the PCA components showed no significant results between play and problem behaviour components. Analysis of PCA components and cat QOL scores showed only the component for stress behaviours (e.g., overgrooming, pica, anxiety) as significantly negatively associated with cat QOL scores (see Tables 3 and 4).

**Table 3.** Results of a one-way analysis of variance (ANOVA) for each factor associated with quality of life in cats (n = 1,590) except for: cat age (n = 1,584) and cat playfulness (n = 1,589). Based on online survey responses of cat guardians between June 22^nd^ and July 17^th^, 2021

Factor	Cat Quality of Life Score	Cat Quality of Life Range	CI (95%)	*P*-value
Cat playfulness				< 0.001
Not very playful, only plays occasionally (478)	68.6	40-80	68.1-69.3	
Neither playful nor not playful (281)	70.8	38-80	70.1-71.5	
Playful, often wants to play (830)	73.3	41-80	72.9-73.7	
Total ‘games’ played^a^				< 0.001
0-1 games (155)	67.8	38-80	66.6-69.1	
2-4 games (856)	71.2	41-80	70.7-71.5	
5-6 games (390)	72.7	40-80	72.2 -73.3	
7+ games (189)	73.5	51-80	72.7-74.2	
Housing^b^				< 0.001
Outdoor access (524)	70.3	41-80	69.7-70.8	
Exclusively Indoors (1,066)	72.1	38-80	71.2-71.8	
Cat age				< 0.001
1-2 years (354)	73.5	50-80	72.9-74.1	
3-5 years (457)	72.3	56-80	71.8-72.8	
6-9 years (377)	71.7	41-80	71.1-72.3	
10+ years (396)	68.5	38-80	67.7-69.3	
Cat health issues				< 0.001
Yes (415)	68.4	38-80	67.6-69.1	
No (1175)	72.6	48-80	72.2-72.8	

a Total ‘games’ played related to games the guardian regularly played with their cat and included: Fetch, playing with catnip toys, playing with noisy toys, playing with boxes, playing with hands, playing with digital devices, playing with wand toys, playing with laser pointers, playing with food, playing with motorised toys, chasing each other and training.

b Housing: Outdoor was defined as regular unsupervised access to the outdoors without a harness, lead and not within a fully enclosed cat enclosure. Indoor/Outdoor was defined as mostly indoor with some access to the outdoors and exclusively indoors was defined as a cat with no access to the outdoors except on a harness or within a fully enclosed cat enclosure.

**Table 4.** General Linear Model parameter estimates of factors associated with cat quality of life scores (n = 1,583), based on online survey cat guardian responses between June 22^nd^ and July 17^th^, 2021

Factor	Coefficient	SEM	Significance
Cat playfulness			< 0.001
Not very playful, only plays occasionally	**		
Neither playful nor not playful	1.2	0.41	
Playful, often wants to play	2.6	0.35	
Total ‘games’ played^a^			0.014
0-1 games	**		
2-4 games	1.2	0.48	
5-6 games	1.5	0.54	
7+ games	1.8	0.62	
Housing^b^			0.018
Outdoor access	**	0.29	
Exclusively indoors	0.68		
Cat age			< 0.001
1-2 years	1.7	0.44	
3-5 years	1.4	0.39	
6-9 years	1.6	0.39	
10+ years	**		
Cat health issues			< 0.001
Yes	**		
No	4.6	0.54	
Stress behaviour score^c^	−0.44	0.07	< 0.001
Cat Owner Relationship Scale (CORS)	0.18	0.02	< 0.001
Interaction: Cat age and health issues			0.005

** Reference category.

a Total ‘games’ played related to games the guardian regularly played with their cat and included: Fetch, playing with catnip toys, playing with noisy toys, playing with boxes, playing with hands, playing with digital devices, playing with wand toys, playing with laser pointers, playing with food, playing with motorised toys, chasing each other and training.

b Housing: Outdoor access was defined as any regular unsupervised access to the outdoors without a harness, lead and not within a fully enclosed cat enclosure. Exclusively indoors was defined as a cat with no access to the outdoors except on a harness or within a fully enclosed cat enclosure.

c Stress behaviour score relates to a guardian-reported frequency for anxiety, pica and overgrooming respectively, reported on a five-point Likert scale and coded into values (1 being never and 5 being most of the time) with values summed to create a composite score.

### Factors associated with cat quality of life

One-way ANOVAs showed that cat QOL scores were higher in cats who were younger, had no health issues, were housed exclusively indoors, were more playful, had access to a greater number of games, had lower stress behaviour PCA component scores, and higher CORS scores (Table 3).

A univariate linear regression showed a significant association between cat QOL and CORS scores (*P* < 0.001). The correlation coefficient for CORS and cat QOL scores was *R* = 0.375, indicating a moderate correlation (standard error = 0.015). A linear regression also showed a significant association between cat QOL scores and stress behaviour PCA component scores (*P* < 0.001). The correlation coefficient for stress behaviours and cat QOL scores was *R* = 0.154 indicating a weak correlation (standard error = 0.08).

A general linear model analysis of factors associated with cat QOL scores showed higher cat QOL scores where cats were housed exclusively indoors, were more playful, had access to a greater number of games, and where the guardian reported higher CORS scores (Table 4).

An interaction was found between reported health issues and cat age (*P* = 0.005). QOL scores reduced at a greater rate where the cat was older and had health issues.

### Factors associated with cat-guardian relationships

One-way ANOVAs showed that CORS scores were higher in human-cat dyads where the cat was housed exclusively indoors, the number of games played within the dyad was greater, the cat had access to a higher number of resources, guardians did not report avoiding play, and where both cat and guardian initiated play (Table 5).

**Table 5.** Results of a one-way analysis of variance (ANOVA) for each factor associated with the cat owner relationship scale (CORS) (n = 1,590) except for: guardian gender (n = 1,582), do you avoid play (n = 1,580), who initiates play (n = 1,451) and relationship status (n = 1,567). Based on online survey cat guardian responses between June 22^nd^ and July 17^th^, 2021

Factor	CORS score	CORS Range	CI (95%)	*P*-value
Total ‘games’ played^a^				< 0.001
0-1 games (155)	105.1	58-130	103.1-107.1	
2-4 games (856)	111.7	80-130	111.1-112.3	
5-6 games (390)	114.3	70-130	113.4 -115.2	
7+ games (189)	115.5	77-130	114.2-116.7	
Who initiates play?				< 0.001
Cat (275)	110.6	76-130	109.4-111.8	
Guardian (313)	110.5	77-130	109.4-111.7	
Both (863)	113.9	70-130	113.3-114.5	
Do you avoid play?				< 0.001
Yes (584)	110.8	70-130	110.1-111.6	
No (996)	112.9	58-130	112.3-113.6	
Housing^b^				< 0.001
Outdoor access (524)	109.7	58-130	108.8-110.6	
Exclusively indoors (1,066)	113.3	70-130	112.7-113.8	
Total resources available^c^				< 0.001
0-3 resources (95)	102.7	58-129	100.1-105.4	
4-6 resources (558)	110.7	73-130	109.9-111.6	
7-9 resources (698)	113.4	70-130	112.7-114.1	
10-14 resources (239)	115.3	77-130	114.2-116.4	
Guardian age				< 0.001
18-24 (179)	115.4	86-130	114.2-116.7	
25-34 (616)	112.5	70-130	111.8-113.3	
35-44 (396)	111.4	76-130	110.4-112.5	
45-54 (245)	111.5	58-130	110.2-112.8	
55+ (154)	109.5	73-128	107.8-111.3	
Guardian gender				< 0.001
Non-binary (52)	115.8	96-130	113.6-118.1	
Female (1,429)	112.3	58-130	111.8-112.8	
Male (101)	107.8	79-126	105.7-109.9	
Relationship status				< 0.001
Single with children (65)	108.8	85-130	105.9-111.6	
Partnered with children (297)	108.3	58-130	106.9-109.6	
Partnered	112.9	73-130	112.3-113.7	
Single	113.6	70-130	112.8-114.5	
Number of hours cat left home alone				0.002
0-10 h (527)	112.7	80-130	111.9-113.5	
11-25 h (420)	111.9	73-130	110.9-112.8	
26-39 h (416)	112.8	58-130	111.8-113.8	
40+ h (227)	109.9	76-130	108.5-111.3	

a Total ‘games’ played related to games the guardian regularly played with their cat and included: Fetch, playing with catnip toys, playing with noisy toys, playing with boxes, playing with hands, playing with digital devices, playing with wand toys, playing with laser pointers, playing with food, playing with motorised toys, chasing each other and training.

b Housing: Outdoor access was defined as any regular unsupervised access to the outdoors without a harness, lead and not within a fully enclosed cat enclosure. Exclusively indoors was defined as a cat with no access to the outdoors except on a harness or within a fully enclosed cat enclosure.

c Total resources available included: scratching post, cat bed, hiding place, water fountain, wand toy, food puzzle, food treats, motorised toy, wall or window perch, harness walks, outdoor cat enclosure, cat grass, self-cleaning litter tray, Feliway^TM^ and automatic feeder.

A univariate linear regression showed significant associations between CORS scores and guardian playfulness (APTS) scores (*P* < 0.001), total guardian-cat daily play time (*P* < 0.001), cat QOL scores (*P* < 0.001), and the length of guardianship. The correlation coefficient for CORS and APTS was *R* = 0.120, indicating a weak correlation (standard error = 0.017). The correlation coefficient for CORS and total guardian-cat daily play time was *R* = 0.271, indicating a moderate correlation (standard error = 0.004). The correlation coefficient for CORS and cat QOL scores was *R* = 0.375 indicating a moderate correlation (standard error = 0.037). The correlation coefficient for CORS and length of guardianship was *R* = 0.058, indicating a weak correlation (standard error = 0.06).

A general linear model analysis of factors associated with CORS scores showed higher scores where cats were housed exclusively indoors, the number of games played within the human-cat dyad was greater, the cat had access to a larger number of resources, guardian age was younger, guardians identified as non-binary, guardians did not report avoiding play, both cat and guardian initiated play, the guardian had higher playfulness scores, were single, and where the cat was left home for less than 40 h (Table 6).

**Table 6.** General Linear Model parameter estimates of factors associated with cat-owner relationship scores (n = 1,372), based on online survey responses of cat guardians between June 22^nd^ and July 17^th^, 2021

Factor	Coefficient	SEM	Significance
Total ‘games’ played^a^			0.034
0-1 games	**		
2-4 games	1.2	0.48	
5-6 games	1.5	0.54	
7+ games	1.8	0.62	
Who initiates play?			< 0.001
Cat	**		
Guardian	0.34	0.72	
Both	2.2	0.62	
Do you avoid play?			< 0.001
Yes	**		
No	2.6	0.47	
Housing^b^			< 0.001
Outdoor access	**		
Exclusively indoors	1.8	0.51	
Total resources available^c^			< 0.001
0-3 resources	**		
4-6 resources	3.8	1.1	
7-9 resources	4.6	1.1	
10-14 resources	5.6	1.3	
Relationship status			< 0.001
Single with children	**		
Partnered with children	0.26	1.2	
Partnered	3.1	1.2	
Single	3.9	1.2	
Number of hours left alone			0.002
0-10 h	2.8	0.73	
11-25 h	1.6	0.75	
26-39 h	2.1	0.75	
40+ h	**		
Guardian age			0.032
18-24	2.3	1.1	
25-34	.51	0.85	
35-44	1.5	0.87	
45-54	1.8	0.93	
55+	**		
Guardian gender			0.002
Male	**		
Female	3.3	0.93	
Non-binary	4.1	1.5	
Adult playfulness trait scale (APTS)	0.04	0.02	0.011
Total cat-guardian daily play	0.03	0.01	< 0.001
Length of guardianship	0.26	0.06	< 0.001
Cat quality of life (QOL) score	0.44	0.04	< 0.001

** Reference category.

a Total ‘games’ played related to games the guardian regularly played with their cat and included: Fetch, playing with catnip toys, playing with noisy toys, playing with boxes, playing with hands, playing with digital devices, playing with wand toys, playing with laser pointers, playing with food, playing with motorised toys, chasing each other and training.

b Housing: Outdoor access was defined as any regular unsupervised access to the outdoors without a harness, lead and not within a fully enclosed cat enclosure. Exclusively indoors was defined as a cat with no access to the outdoors except on a harness or within a fully enclosed cat enclosure.

c Total resources available included: scratching post, cat bed, hiding place, water fountain, wand toy, food puzzle, food treats, motorised toy, wall or window perch, harness walks, outdoor cat enclosure, cat grass, self-cleaning litter tray, Feliway^TM^ and automatic feeder.

### Guardian-perceived behaviour changes when play is withheld

Guardians were presented with the following question: “Does your cat’s behaviour change if you haven’t played with them for a while?” The most common reported behaviour changes when play was withheld (n = 468) were that their cat exhibited more attention-seeking behaviour, followed by increased vocalising, an increase in destructive behaviour, an increase in reclusive behaviour, and increased aggressive behaviour (Table 7). Examples of quotes relating to these reported behaviours are given in Table 8.

**Table 7.** Guardian-reported cat behaviour changes when play was withheld, reported by guardians (n = 468). Guardians could report more than one behaviour change. Based on online survey responses between June 22^nd^ and July 17^th^, 2021

Behaviour change associated with lack of play	*n*	%
Increased attention seeking behaviour	104	22.2%
Increased vocalising	73	15.6%
Reclusive behaviour	49	10.5%
Increased destructive behaviour	47	10.0%
Increased aggressive behaviour	43	9.0%
Appearing grumpy	34	7.3%
Restlessness	28	6.0%
Increase in time spent sleeping	17	3.6%
More likely to initiate play/bring toys to guardian	16	3.4%
Increased ‘zoomies’	16	3.4%
Re-directs playfulness to household cats	15	3.2%
Appears to be ‘sad’	14	3.0%
More likely to interrupt guardians sleep time	13	2.8%

**Table 8.** Examples of guardian-reported cat behaviour changes when play was withheld (n = 468). Based on online survey responses between June 22^nd^ and July 17^th^, 2021

Example	Quote
Attention-seeking behaviour	*“He behaves to get my attention; does things he knows he’s not supposed to, e.g., jumping on top of cabinets.” [participant 729]*
Destructive behaviour	*“They are frustrated and destroy property (curtains, couch etc)” [participant 178]*
Increased vocalisation	*“He will start screaming at night while carrying toys up the stairs to my bedroom” [participant 864]*
Reclusive behaviour	*“My cat tends to not engage with us as much. She’ll spend time around us, but she’s more aloof. As soon as we take her out for a walk or play with her, her behaviour changes the following day and she’s very playful and affectionate”. [participant 683]* *“She becomes more distant from me…doesn’t sleep near me as much…is less accepting of affection.” [participant 677]*
Increased aggressive behaviour	*“He gets frustrated and takes it out on myself or the other cat (aggressive, violent play.”) [participant 1459]*

## Discussion

The objective of the present study was to investigate associations between play and welfare in cats by assessing factors associated with cat QOL score and closeness of cat-guardian relationships. Results indicated that cat playfulness and the number of games played by the cat and guardian were associated with QOL scores while the amount of daily play, the number of games played, who initiated play, whether the guardian avoided play, and guardian playfulness were associated with cat-guardian relationship scores. Cat behaviour, as perceived by their guardian, was reported to change during times when play was withheld. No significant associations were found between play and problem behaviours.

### Play factors associated with cat quality of life: Cat playfulness and number of games

Cat playfulness was significantly correlated with higher cat QOL scores. Previous observations suggest that play is more likely to be exhibited during times when animals are physically healthy, have access to ample resources, and are not under stress (Held & Špinka 2011; Ahloy-Dallaire *et al*. 2018). Cat playfulness may therefore be reflective of how a cat is feeling physically or emotionally, with sick, injured or stressed cats being less likely to exhibit playfulness (Fagen 1978). In a previous study into the effects of stress on cats, animals assigned to a stress condition of irregular or poor caretaking showed reduced or absent play behaviours (Carlstead *et al*. 1993). Interestingly, contrary to our expectations, daily play times were not significantly associated with cat QOL scores. However, the higher the number of games a cat was reported to regularly engage in was significantly correlated with higher cat QOL scores. This discrepancy may indicate that it is the quality or variety of play available and not simply the quantity of play that may be associated with positive welfare outcomes for cats (Carlstead *et al*. 1991; Ahloy-Dallaire *et al*. 2018; Fernandez 2022).

The following distinction was made in this study between toys and games: toys were defined as stimuli that the cat can interact with while games were defined as actions that a cat (and/or guardian) can take. A game may include a toy. Cats have previously been observed to become habituated to toys quickly, showing decreases in play intensity with increased exposure to a particular toy followed by regaining interest and intensity of play when presented with a new toy (Hall *et al*. 2002). Cats may also become habituated to the types of games available to them. Where cats only had a few games available to them with their guardian, their guardian also reported lower QOL scores. If cats are given access to a greater number or regular variation of games and toys, this may help to minimise habituation and boredom with play, potentially resulting in higher playfulness, QOL scores and overall welfare.

### Play factors associated with cat-guardian relationships: Number of games and daily play

As well as being associated with cat QOL scores, a higher number of games regularly engaged with was also associated with higher cat-guardian relationship scores. It is possible that guardians who report having a low CORS score were less likely to interact with their cat, or that regular engagement in a variety of play is beneficial for improving cat-guardian relationships. Similarly for humans, only engaging in a small number of games with their cats may lead guardians to experience boredom and dissatisfaction with play, potentially resulting in guardians playing less with their cats. It has previously been found that routine behaviours, which a person feels obligated to perform, undermine their sense of self and freedom (Iso-Ahola 2022). However, these negative aspects can be mitigated by increasing the variety in, and of, these behaviours, such as undertaking a variety of different activities that achieve the same purpose (Iso-Ahola 2022). Tasks that require too little cognitive engagement (such as repeating the same wand toy game with a cat) also can become boring and aversive, leading people to either disengage with the task or seek to enhance variety and novelty (Shenhav *et al*. 2016). Therefore, engaging with a variety of games is likely to increase both cat and guardian feelings of satisfaction and engagement with their play sessions, enhancing the human-cat bond and making future play sessions more likely. Henning *et al*. (2022b) previously reported that the number of games engaged with by human and cat was also associated with longer play session lengths and total daily play times. Total daily play times were significantly associated with cat-guardian relationship scores. This may be because more play offers more opportunities for human-cat bonding, or it may also be that guardians who are more invested in their cat are more willing to play with them.

### Play factors associated with cat-guardian relationships: Who initiates and guardian playfulness

Cat-guardian relationship scores were highest where both cat and guardian were reported to initiate play sessions. Being able to both initiate play and recognise initiation of play requires both cat and guardian to be observant and capable of comprehending each other’s communication signals. Communication is key to the health of many relationships; therefore, it is possible that better understanding of human and cat signals, be they vocalisations or body language, is likely to lead to a closer or more enriching cat-guardian relationship. Guardians who are able to understand their cat’s signals are also more likely to identify their cat’s needs (Heath 2007). Self-determination, including the freedom to choose to initiate and engage in an activity, is also a critical component to enjoyment in activities (Dattilo *et al*. 1998). Therefore, when a cat or guardian is choosing to participate in an activity, instead of being forced to do so, they may be more likely to derive benefit from the activity. Cats have been observed to respond well to being given choice and control within human-cat interactions (Mertens & Turner 1988; Haywood *et al*. 2021). Allowing cats to have choice in their interactions is an important behaviour to encourage within cat guardians. Further, cats have been shown to engage more enthusiastically with humans who are more responsive when the cat is seeking attention (Turner 1991). If a cat is regularly initiating play sessions, it indicates that they are applying, and able to utilise, their own self-determination.

Guardian playfulness was also found to be significantly associated with higher cat-guardian relationship scores. Guardians who are more playful may be more willing to engage in play with their cat and may enjoy playing with their cat more than guardians who are not playful. Henning *et al*. (2022b), which utilised data from the same respondents as the current study, previously found that APTS scores were significantly associated with longer play session times between cats and guardians. A more engaged guardian who participates in longer play sessions may be better positioned to build social connections with their cat through increased interaction. In a previous study by Odendaal and Meintjes (2003) into dog-guardian relationships, interactions of between 5 and 24 min were associated (for both dog and human) with: decreases in blood pressure; significant increases in oxytocin, prolactin and phenylethylamine (neurochemicals that are known to be associated with bonding), plasma dopamine concentrations associated with pleasurable experiences and β-endorphin which is involved in learning, memory, analgesia, and euphoric states. Humans in the study also experienced a decrease in plasma cortisol, indicating stress relief (Odendaal & Meintjes 2003). Increased interactions due to guardian playfulness may therefore benefit both cat and guardian.

However, it is important to note that this measure of cat-guardian relationship is solely guardian-reported and is not a direct measure of how the cat experiences the relationship. A recent study by Finka *et al*. (2022) found that cats preferred humans who interacted only when the cat showed interest in interacting and that people who self-reported as having a long history of experience with cats were more likely to force interaction, hold cats against their will and touch places that cats do not typically like to be touched (Finka *et al*. 2022). Direct observational measures of cat experiences of cat-guardian relationships are needed.

### Play factors associated with cat quality of life and cat-guardian relationships: Indoor or outdoor housing

Exclusively indoor housing was significantly associated with both higher guardian-reported cat QOL scores and higher cat-guardian relationship scores. To our knowledge, this is the first study to compare QOL scores of indoor- and outdoor-housed cats. Some previous studies have focused on cat adaptation to confinement in cattery, shelter, or laboratory settings. However, these are limited in their ability to assess the needs and welfare of cats within a long-term, indoor-only home (Ottway & Hawkins 2003; Kry & Casey 2007; Stella *et al*. 2014; Rehnberg *et al*. 2015; Foreman-Worsley & Farnworth 2019). It has been suggested that outdoor cats may benefit from higher quality of life generally than indoors cats due to their ability to find their own amusement, express their natural behaviours, and choose whether to be inside or out (Rochlitz 2005). For some, this potential for a higher quality of life outweighs the increased risks of outdoor access, such as infectious disease, road accidents, trauma, and risk to native wildlife (Yeates & Yates 2017). While for others, the inherent dangers of the outdoors and their potential to limit the length of their cat’s life outweigh any small difference in potential quality of life (Rochlitz 2005). However, contrary to the suggestion that outdoor cats may have a higher quality of life, the results of the current study showed that cats housed exclusively indoors recorded higher QOL scores than cats with outdoor access. While this is not a definitive result regarding the difference in QOL scores between indoor and outdoor cats, there are several reasons this result may have been observed in our study. Firstly, cat-guardian relationships are central to domestic cat welfare. Cats rely heavily on their guardian to care for their nutrition, shelter and both their physical and mental health. A good relationship with their guardian, therefore, is likely to increase their QOL score. Our results found a significant association with cat-guardian relationship scores and whether the cat was housed exclusively indoors or allowed outdoor access, with indoor cat guardians reporting higher cat-guardian relationship scores. Secondly, guardians who house their cats indoors are better able to observe their cat’s day-to-day health. Any changes in behaviour, mobility, or important health markers, such as urination and defaecation regularity and consistency, are more likely to be noticed by a guardian who is observing their cat more often. Conversely, cats with outdoor access may be harder to observe and may eliminate in outdoor areas where their excrement cannot be examined for changes. If indoor-cat guardians are more able to observe changes in their cat’s health, they are better placed to make any environmental changes needed, or seek veterinary attention, resulting in increased cat QOL scores. Finally, guardians who keep their cats indoors may have more opportunities to participate in enriching interactions such as play. A previous study by Pyari *et al*. (2021) reported that indoor cats showed stronger interest in play stimuli than outdoor cats. It is possible then, that indoor cat play preferences and needs may differ from outdoor cats. Pyari *et al*. (2021) discussed that this difference may be due to an absence of the ability to express hunting behaviour in indoor cats, leading to what they describe as an increase in predatory play behaviour to compensate. However, predatory play as a term and as a behaviour is not yet fully understood and may in fact be a misnomer. A previous study by Pellis *et al*. (1988) analysed ‘predatory play’ movements or playful movements during predation and found that these movement patterns were adaptive movements that functioned to protect the cat from injury during predation and in essence were not playful at all (Pellis *et al*. 1988). The trope of cats being ‘psychopathic’ playful hunters (Evans *et al*. 2021) is a potentially dangerous one for cat welfare, considering the current attitudes to cats and their frequent use as a scapegoat for the more major pressures imposed by people on biodiversity and climate (Palmer 2014; Wald & Peterson 2020). It is important that we acknowledge the play needs of cats and that these needs may differ depending on that cat’s background and housing, while not anthropomorphising and casting moral judgments that do not apply to non-human animals and may not be an accurate assessment of their behaviour anyway, as observed here by Pellis *et al*. (1988).

While there are strong arguments for both camps of thought as to whether cats should be housed indoors or out, it is generally agreed that indoor cats require environmental enrichments to support their welfare in an indoor only home (Foreman-Worsley & Farnworth 2019). It is also possible that guardians who are more closely bonded with their cat and therefore may be more worried for their cat’s safety may choose to house their cats indoors (Crowley *et al*. 2019, 2020; Foreman-Worsley *et al*. 2021) (as indicated here by the higher cat-guardian relationship scores), and that they may also be more willing to fulfill the play and enrichment needs of their cat. This, in turn, may have resulted in the higher cat QOL scores observed within this study. Further, it may not be that indoor or outdoor housing is strictly better for cats, but instead, how their guardians perceive, monitor, and treat them that has more of an impact.

### Behaviour changes during lowered play times

Guardians reported noticing certain behaviour changes when they had not played with their cat for some time. While many guardians reported their cat exhibiting an increase in attention-seeking behaviours, several reported that their cat became more reclusive when play had been withheld. This showcases an interesting dichotomy of reactions to lack of play, with both an increase and decrease in cat engagement with guardian observed depending on the individual cat. This may be due to individual differences between cats and/or their guardians, familial dynamics within the household, the specific environment, or the housing situation of the cat (indoor or outdoor). Unfortunately, no clear pattens of differences were able to be deduced between behaviour changes in indoor versus outdoor cats within this study, however it is likely that this may impact behaviours and future studies could investigate this further. For many of the welfare measures we have investigated previously, the emphasis has been on whether play is indicative of welfare (Henning *et al*. 2022a). Observations of behaviour change when play is withheld may also offer us an insight into whether play is a promotor of welfare. Often it is difficult to separate the two concepts from each other, not knowing if more play means more welfare or if greater welfare means more play, or both. Within this study, guardians reported that when play is withheld, observable behaviour changes occur that may indicate a decrease in welfare or well-being. Increased attention-seeking behaviour, especially vocalisations and destructive behaviour, may indicate that the cat is experiencing frustration, a negative affective state that may impact the cat’s well-being. Increased reclusive behaviour may also be detrimental to cat welfare. Cats in human homes must share their space with the humans of the household, who utilise most of the space in a house. A cat who becomes reclusive is limited in what areas of the house they feel comfortable accessing and this may limit their physical activity as well as their access to important resources such as food, water, and litter (Carlstead *et al*. 1993). Reclusive behaviour may also indicate that there has been a deterioration in the social relationship between cat and guardian. Within cat social groups, signs of affiliation include spending time around and interacting positively with another cat while avoidance of spaces where another cat is and aversion to interacting may indicate that the cats are not affiliated (Vitale 2022). Put in terms of cat and guardian, if a cat is avoiding spaces where their guardian is and is not interacting as usual, it may indicate that the cat is uncomfortable. Humans within relationships are often acutely attuned to the behaviour of those close to them, and how this behaviour may affect or be indicative of the health of their relationship. It is possible that cats are similarly aware of their guardian’s behaviour towards them and what this may indicate about their safety within the relationship. Recent research shows that cats regularly keep a mental tab on where their guardian is within the house (Takagi *et al*. 2021). It is entirely possible that cats are also aware of what constitutes their guardian’s regular behaviour and therefore notice any changes to this behaviour. Previous studies have shown that cats are highly sensitive to changes in routine (Stella *et al*. 2011). It is possible that changes the cat perceives in their regular interactions with their guardians may similarly make them uncomfortable. Many of the behaviour changes listed, such as destructive or aggressive behaviour, may also constitute a problem or annoyance for the guardian which may impact their perception of their relationship with their cat. As cat welfare within human homes relies so heavily on human perceptions, this may also constitute an impact to cat welfare.

### Limitations and future research

This study has several potential limitations. Since it is not a direct measure, guardian-reported surveys are inherently limited in their ability to accurately capture their animals’ behavioural data. Further, due to the self-reporting nature of surveys, an individual’s responses may be prone to respondent and recall bias and limited in their ability to predict or assess behaviour (Schwarz & Oyserman 2001; Paulhus & Vazire 2007; Kormos & Gifford 2014). In addition, the survey population sampled may also be biased. Participants were recruited during a global pandemic and overwhelmingly identified themselves as female; the survey also took approximately 20–30 min to complete, and guardians prepared to volunteer for a survey of this length may be more invested in their cat’s care than the average cat guardian. Therefore, survey responses may not be an accurate representation of the general population. Due to some overlap between the validated scales and questions such as frequency of stress and other problem behaviours, findings between the two may be limited in their ability for meaningful comparison. Finally, as this was a cross-sectional study it does not prove a causal link between factors that were associated.

Future studies should further investigate the role of play in cat welfare with special attention paid to indoor and outdoor requirements, the mechanisms of how play impacts cat-guardian relationships, communication, socialisation, and play preferences in both cats and guardians.

## Animal welfare implications and conclusion

Play has potential to be used as both an indicator and promotor of cat welfare. The present study aimed to assess the play factors associated with welfare in cats. Within this study, multiple welfare-impacting measures were used, including cat QOL score, the quality of the relationship between cat and guardian, the prevalence of problem behaviours and observations of behavioural changes. Results showed significant associations between cat playfulness and the number of games played with cat QOL scores. Cat-guardian relationship scores were significantly associated with the amount of daily play and number of games engaged in, whether the cat and guardian both initiated play sessions and guardian playfulness. Problem behaviours that clustered on a PCA into a stress component were significantly associated with QOL scores, but no other problem behaviours were found to have significant associations with play or welfare measures. Behavioural changes that indicated stress, frustration, or unease were reported when play had been withheld. This study shows support for the association between play and welfare and provides several avenues for further research. Future investigations should focus on the role of play in assessments of cat welfare, the use of play as an intervention to promote welfare, and the mechanisms of how play impacts cat welfare and cat-guardian relationships.
